# Wrinkled Interfaces: Taking Advantage of Anisotropic Wrinkling to Periodically Pattern Polymer Surfaces

**DOI:** 10.1002/advs.202207210

**Published:** 2023-02-12

**Authors:** Ning Liu, Qichao Sun, Zhensheng Yang, Linna Shan, Zhiying Wang, Hao Li

**Affiliations:** ^1^ National‐Local Joint Engineering Laboratory for Energy Conservation of Chemical Process Integration and Resources Utilization School of Chemical Engineering and Technology Hebei University of Technology Tianjin 300130 China

**Keywords:** oriented wrinkle, periodically patterned surfaces, self‐organization, surface instabilities, surface patterning techniques, wrinkling

## Abstract

Periodically patterned surfaces can cause special surface properties and are employed as functional building blocks in many devices, yet remaining challenges in fabrication. Advancements in fabricating structured polymer surfaces for obtaining periodic patterns are accomplished by adopting “top‐down” strategies based on self‐assembly or physico‐chemical growth of atoms, molecules, or particles or “bottom‐up” strategies ranging from traditional micromolding (embossing) or micro/nanoimprinting to novel laser‐induced periodic surface structure, soft lithography, or direct laser interference patterning among others. Thus, technological advances directly promote higher resolution capabilities. Contrasted with the above techniques requiring highly sophisticated tools, surface instabilities taking advantage of the intrinsic properties of polymers induce surface wrinkling in order to fabricate periodically oriented wrinkled patterns. Such abundant and elaborate patterns are obtained as a result of self‐organizing processes that are rather difficult if not impossible to fabricate through conventional patterning techniques. Focusing on oriented wrinkles, this review thoroughly describes the formation mechanisms and fabrication approaches for oriented wrinkles, as well as their fine‐tuning in the wavelength, amplitude, and orientation control. Finally, the major applications in which oriented wrinkled interfaces are already in use or may be prospective in the near future are overviewed.

## Graphical abstract

Advances in the fabrication of periodic patterns on polymer surfaces taking advantage of anisotropic wrinkling are summarized. This review thoroughly describes the formation mechanisms and fabrication approaches for oriented wrinkles, as well as their fine‐tuning in the wavelength, amplitude, and orientation control. Finally, the major applications in which oriented wrinkled interfaces are already employed or may be prospective are overviewed.

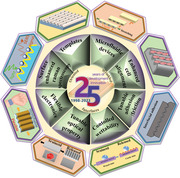

## 1 Introduction

Periodically patterned polymer film surfaces have been used as functional building blocks in many devices yet challenges remain in their fabrication during the last decades evidenced by the numerous applications generated from them.^[^ ^1^, ^2^, ^3^, ^4^ ^]^ Periodic patterning of polymer films serves great functional purposes for applications ranging from optical elements,^[^ ^5^, ^6^ ^]^ microfluidic devices,^[^ ^7^, ^8^ ^]^ flexible and stretchable electronics^[^ ^9^, ^10^ ^]^ as well as templates for constructing complex hierarchical structures^[^ ^11^, ^12^ ^]^ just to mention few of them.

The advancements in fabricating periodically patterned surfaces have been accomplished through two strategies, mainly classified as “bottom‐up” and “top‐down.” The former is based on the self‐assembly or physico‐chemical growth of atoms, molecules, or particles to construct ordered structures on micron or even nanometer scale. The dimension and morphology of the structures obtained are adjustable within a certain range and have the advantages of low cost, convenient operation, and broad applicability. While such a strategy allows reaching smaller periods, it is more prone to defects and has limitations in pattern control. Besides specific periods require the synthesis of specialized compounds.^[^ ^13^, ^14^, ^15^, ^16^, ^17^ ^]^ The latter adopts traditional techniques, such as micromolding (embossing)^[^ ^18^, ^19^, ^20^, ^21^, ^22^ ^]^ or micro/nanoimprinting,^[^ ^23^, ^24^, ^25^, ^26^ ^]^ and/or by developing novel techniques with higher resolution capabilities including laser‐induced periodic surface structure,^[^ ^27^, ^28^ ^]^ soft lithography,^[^ ^29^, ^30^, ^31^, ^32^ ^]^ or direct laser interference patterning,^[^ ^33^, ^34^ ^]^ among others. Technological advances offer unparalleled versatility and fidelity. However, they face inherent limitations in terms of lateral periodicities and are generally rather expensive. Furthermore, both strategies face serious difficulties in scaling up to macroscopic dimensions.^[^ ^35^ ^]^

In contrast to the above‐mentioned techniques limited by highly sophisticated tools and macroscopic dimensional scale‐up, surface instabilities taking advantage of the intrinsic properties of polymers induce surface wrinkling in order to fabricate periodically oriented wrinkled patterns. Such abundant and elaborate patterns obtained as a result of self‐organizing processes are often generated spontaneously from within the system during polymer film processing. Crucially, it allows creating topographic patterns with periodicities between few 100 nm and many hundreds of microns and has few restrictions concerning upscaling.^[^ ^10^, ^35^, ^36^, ^37^, ^38^, ^39^, ^40^ ^]^

In this review, we focus on providing a comprehensive overview of controlled wrinkling, with an emphasis on oriented wrinkle formation. To the best of our knowledge, the most recent advances in this field have only been reviewed to a partial extent. Researchers prefer to concentrate on the fabrication approaches of oriented wrinkles, as well as the analysis and control of wrinkle orientation via experimental parameters.^[^ ^41^, ^42^, ^43^ ^]^ Other reviews have not reported on the most recent advances in this rapidly developing field.^[^ ^44^, ^45^, ^46^ ^]^ Here, we will present an updated overview of this field, providing a reference point for those researchers interested in the fabrication of functional and multistructural surfaces with periodic patterns.

For that purpose, we will introduce oriented wrinkles on the background of surface instability, with the first objective of briefly defining oriented wrinkles as well as describing their categories (Section 2). Typically, wrinkles obtained by instability‐mediated techniques are randomly distributed. Therefore, the formation mechanisms of oriented wrinkles, including both mechanical and physical bases, will be thoroughly analyzed in detail in Section 3. Section 4 will be devoted to their fabrication techniques, dimensional fine‐tuning as well as orientation control, which include film wrinkling systems composed of layered materials with mismatched mechanical properties and materials with gradually variable mechanical properties; the force stimulus employed to induce wrinkling; the main parameters that have to be taken into account for providing control over the wrinkle dimensions, orientation, and their final morphology in different film wrinkling systems; lastly, a particular focus will be provided on more sophisticated systems in which the formation of complex patterns with tunable/stimulus‐responsive will be covered. Finally, the main areas in which oriented wrinkled interfaces have been applied or may be expected to be employed are overviewed in Section 5. Their use as templates, flexible electronics, supports with tunable optics or adhesion, cellular arrangements, microfluidic devices, or for catalysis‐related applications are a few of the areas in which oriented wrinkled interfaces are likely to be exceptionally appealing.

## 2 What Are Oriented Wrinkles?

Surface wrinkling is a common instability phenomenon occurring in polymeric films, generally occurring at the mesoscale where molecular horizontal forces predominate or in highly confined systems (active interaction between the polymer interfaces) which are by definition unstable.^[^ ^24^, ^45^, ^47^ ^]^ Studies have indicated that the wrinkling phenomenon and the resulting morphology are intrinsically the direct results of the competition between destabilizing and stabilizing forces within the films.^[^ ^24^, ^43^ ^]^ Typically, metastable films are susceptible to becoming unstable when stimulated by mechanical stresses (heating, stretching, or osmotic pressure); upon removing the external stimulus, wrinkling occurs and the film reaches a new metastable situation.^[^ ^45^, ^48^ ^]^

In general, several wrinkle arrays with repeating identical dimensions organize along one or several independent directions to produce long‐range ordered periodic wrinkles. Such periodic wrinkles are described as “oriented wrinkles” and present a highly regular arrangement.^[^ ^49^, ^50^, ^51^, ^52^ ^]^ In terms of wrinkle distribution, other wrinkles without the periodic characteristic are disordered, they do not have any preferential orientation and display a random distribution.^[^ ^53^, ^54^, ^55^ ^]^

**Figure 1:**
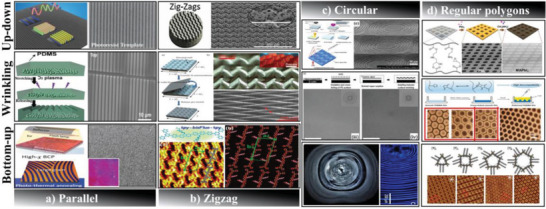
Periodic patterns fabricated by different strategies. Parallel: a) Photoresist templates prepared by nanoimprint lithography. Adapted with permission.^[^ ^65^ ^]^ Copyright 2015, John Wiley & Sons Inc. Wrinkled surface prepared upon mechanical stretching and releasing. Adapted with permission.^[^ ^56^ ^]^ Copyright 2018, John Wiley & Sons Inc. Directed self‐assembly in practical nanolithography. Adapted with permission.^[^ ^66^ ^]^ Copyright 2017, John Wiley & Sons Inc. Zigzag: b) Patterns obtained by photolithography and replica molding. Adapted with permission.^[^ ^67^ ^]^ Copyright 2019, Elsevier. Origami patterns upon releasing the prestretch. Adapted with permission.^[^ ^68^ ^]^ Copyright 2016, John Wiley & Sons Inc. Nanopatterning of photoactive precursors by self‐assembly. Adapted with permission.^[^ ^69^ ^]^ Copyright 2022, American Chemical Society. Circular: c) Electron beam lithography and UV‐nanoimprint lithography. Adapted with permission.^[^ ^70^ ^]^ Copyright 2022, Elsevier. Symmetric wrinkling patterns upon solvent swelling. Adapted with permission.^[^ ^61^ ^]^ Copyright 2009, John Wiley & Sons Inc. Self‐assembly of concentric microrings. Adapted with permission.^[^ ^71^ ^]^ Copyright 2020, Elsevier. Regular polygons: d) Patterned by a photolithographic process using a shadow mask. Adapted with permission.^[^ ^72^ ^]^ Copyright 2019, American Chemical Society. Patterning of PHEMA hydrogel films upon swelling. Adapted with permission.^[^ ^49^ ^]^ Copyright 2021, American Chemical Society. Nanopatterning by molecular polygons.Adapted with permission.^[^ ^73^ ^]^ Copyright 2011, American Chemical Society.

In terms of shape, oriented wrinkles can be further subdivided, typically including parallel,^[^ ^56^, ^57^, ^58^ ^]^ zigzag,^[^ ^10^, ^59^, ^60^ ^]^ circular,^[^ ^61^, ^62^ ^]^ and regular polygons (tetragonal, hexagonal, etc.).^[^ ^49^, ^63^, ^64^ ^]^ We have compared these patterns with the periodic patterns fabricated by current patterning techniques, as illustrated in **Figure** **1**.

The comparison result reveals that, except for particular periodic structures like micropillar arrays,^[^ ^22^, ^23^, ^74^, ^75^, ^76^ ^]^ the wrinkling technique has been almost capable of fabricating periodic patterns with variable shapes and dimensions as produced by conventional techniques or high precision techniques. Therefore, a thorough understanding of the theories as well as the parameters that affect surface wrinkling provides a promising alternative for obtaining unprecedented periodic patterns and surface morphologies.

## 3 Mechanisms of Oriented Wrinkle Formation

### 3.1 Wrinkling Mechanism

**Figure 2:**
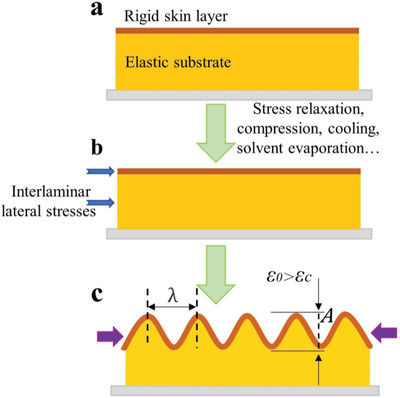
Schematic diagram of wrinkling model composed of an elastic substrate as well as a rigid skin layer attached on top of it.

The generally accepted wrinkling model was first defined by professor Rodríguez‐Hernández and he proposed comprehensive wrinkling mechanisms, as depicted in **Figure** **2**. This model is usually composed of an elastic substrate as well as a rigid skin layer attached on top of it (Figure 2a),^[^ ^45^, ^77^, ^78^, ^79^, ^80^ ^]^ the skin and elastic substrate have significantly dissimilar elastic modulus and Poisson's ratio. Any external force stimulus (thermal, mechanical stretching/compression, swelling…) capable of inducing a stress exceeding a critical value will result in a mismatch of mechanical properties between the skin and the substrate.^[^ ^47^, ^81^, ^82^ ^]^ As a result, interlaminar lateral stress along the plane generates (Figure 2b), causing a compressive stress *F* in the capping skin at the same time.^[^ ^53^, ^80^, ^83^ ^]^

*F* can be calculated by using the following expression^[^ ^45^, ^47^, ^79^ ^]^

(1.1) $$F=E_{s}\;\left[{\left(\frac{\pi }{\lambda }\right)}^{2}\frac{wh^{3}}{3\left(1-\nu_{s}^{2}\right)}+\frac{\lambda }{4\pi }\frac{E_{f}w}{\left(1-\nu_{f}^{2}\right)E_{s}}\right]$$

where *h* and *w* refer to the thickness and width of the skin, respectively; *λ* refers to the wavelength of the wrinkle; *E*_s_ and *E*_f_ refer to the elastic modulus of the skin and substrate, respectively; *ν*_s_ and *ν*_f_ refer to the Poisson's ratio of the skin and substrate, respectively.

Loading exceeding the buckling limit (i.e., the critical value *F*_c_) will induce the skin to buckle, forming wrinkles to release the interlaminar lateral stress for maintaining a stable state. When the buckling has just started, *dF*/*dλ* = 0. Combined with Equation (1.1), the calculation equation of the theoretical wavelength for wrinkles can be obtained as follows^[^ ^46^, ^77^, ^78^, ^79^, ^84^, ^85^ ^]^

(1.2) $$\lambda_{c}=2\pi h{\left[\frac{(1-\nu_{f}^{2})E_{s}}{3(1-v_{s}^{2})E_{f}}\right]}^{1/3}$$

From Equation (1.2), *λ* depends only on the thickness of the skin and the material properties of the skin and substrate.^[^ ^83^, ^86^ ^]^ The calculation equation of the theoretical amplitude *A*c for wrinkle can be obtained as follows

(1.3) $$A_{c}=h{\left(\frac{\varepsilon_{0}}{\varepsilon_{c}}-1\right)}^{1/2}$$

where *ε*_c_ and *ε*_0_ refer to the critical wrinkling strain and applied strain, respectively. When *ε*_0_ > *ε*_c_, surface wrinkling occurs (Figure 2c). The theoretical critical wrinkling strain can be calculated by the following equation^[^ ^87^, ^88^, ^89^, ^90^ ^]^

(1.4) $$\varepsilon_{c}=-\frac{1}{4}{\left(\frac{3{\bar{E}}_{s}}{{\bar{E}}_{f}}\right)}^{2/3}$$

where $\bar{E}$  =  *E*/(1 − *ν* ^2^) refers to the plane strain modulus. The critical wrinkling strain, according to Equation (1.4), is associated with the difference in physical properties between the skin and the substrate materials. The greater the difference in physical properties, the lower the critical wrinkling strain required, which facilitates wrinkle generation.

Later, Cerda et al. also proposed a prediction formula for the wavelength and amplitude based on the minimum energy principal theory, i.e., the wavelength and amplitude are the result of minimizing the total energy (*U*), where *U*  =  *U*_B_ + *U*_S_ (the bending energy of the skin (*U*_B_) and the stretching energy of the elastic substrate along the wrinkles (*U*_S_)).^[44,^ ^79^, ^91^ ^]^ The derived equations for calculating the wavelength and amplitude are given below

(1.5) $$\lambda ∼{\left(\frac{B}{K}\right)}^{1/4}$$

(1.6) $$A∼{\left(\frac{\Delta }{w}\right)}^{1/2}\lambda$$

where *B* refers to the bending stiffness of a thin sheet, *K* refers to the stiffness for an effective elastic substrate, and Δ/*W* refers to an imposed compressive strain. Except for planar films, this prediction equation is also applicable to calculate the wrinkle parameters for other geometries.^[^ ^44^, ^91^, ^92^ ^]^

### 3.2 Orienting Mechanism

**Figure 3:**
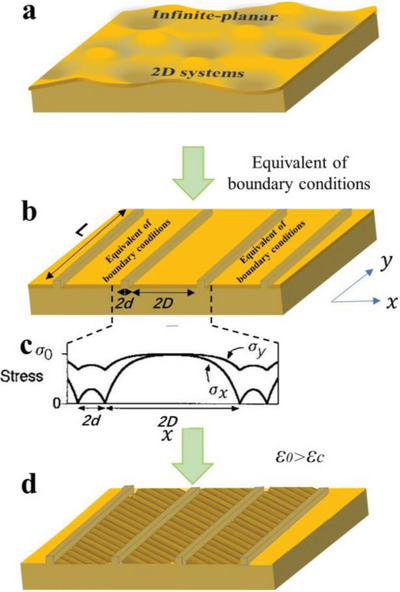
Anisotropic wrinkling model for oriented wrinkle generation. Adapted with permission.^[^ ^95^ ^]^ Copyright 1998, Springer Nature.

The direction of the wrinkles at one point on the layer is perpendicular to the interlaminar lateral stress.^[^ ^53^, ^83^, ^84^ ^]^ When the lateral stress induces surface morphology changes within a skin/substrate film wrinkling system, the dimension of the system can be considered as an infinite‐planar 2D system compared with the wavelength for the surface corrugation (**Figure** **3**a).^[^ ^53^, ^92^ ^]^ In such a system, wrinkles do not have any preferential orientation since the direction of the in‐plane compressive stress in the capping skin is expected to be, on average, isotropic. Therefore, wrinkles are disordered with isotropic interlaminar lateral stress.^[^ ^53^, ^83^, ^93^, ^94^ ^]^ This argument implies that, if anisotropy to the interlaminar lateral stress is deliberately or otherwise introduced, the resulting wrinkle patterns should appear anisotropic characteristics.

The framework of the Allen model originally provided the theoretical basis for anisotropic wrinkling.^[^ ^96^ ^]^ Subsequently, the universally recognized mechanism of oriented wrinkle formation was first proposed by Bowden et al., and they developed an anisotropic wrinkling model that relates the spatially nonuniform lateral‐stress distribution within the skin on a patterned substrate to the wrinkle patterns, as depicted in Figure 3. This model provides reliable estimates of the circumstances in which wrinkles emerge, as well as their wavelength and orientation.^[^ ^95^ ^]^

When equivalent boundary conditions are introduced, here the substrate surface is patterned into bas‐relief structures as physical constraints, where the length, width, and periodic interval of the relief structures are *L*, 2*d*, and 2*D*, respectively, and *L*>>*d*, *D*, as shown in Figure 3b. In this case, the compressive stress in the capping skin will no longer be, on average, isotropic. There is a strong orientation to the compressive stress in the vicinity of the reliefs, with an associated maximum principal compressive stress direction at each point. The wrinkles develop with crests aligned perpendicular to the direction of maximum compressive stress.^[^ ^95^ ^]^

The predictability of the wrinkle patterns is demonstrated by analysis of an array of long straight reliefs parallel to the *y*‐axis, width 2*d*, separated by 2*D*. Since the substrate offers little resistance to displacement of the capping skin in the direction perpendicular to the step, this displacement relieves the stress in that direction, becoming zero at the step of the reliefs (Figure 3c). For the relief itself, the stress distribution in the capping skin before wrinkling can be expressed by Equations (1.7) and (1.8).^[^ ^36^, ^95^ ^]^ The transition length *l* (m) characterizing the distribution of stress from the step to the remote smooth capping skin is given by Equation (1.9)

(1.7) $$\sigma_{x}=-\sigma_{0}\left[1-\frac{\cosh\left(x/l\right)}{\cosh\left(d/l\right)}\right]$$

(1.8) $$\sigma_{y}=-\sigma_{0}\left[1-\nu_{s}\frac{\cosh\left(x/l\right)}{\cosh\left(d/l\right)}\right]$$

(1.9) $$l\approx 0.3h\left[\frac{E_{s}\left(1-\nu_{f}^{2}\right)}{E_{f}\left(1-\nu_{s}^{2}\right)}\right]$$

where *σ*_*x*_ (Pa) is the stress in the *x*‐direction, *σ*_*y*_ is the stress in the *y*‐direction, *σ*_0_ is a uniform, isotropic compressive stress within the same system (see ref. [95] for details), and *x* is measured from the center of the relief (|*x*|<*d*) (Figure 3c).

The above equation is also applicable to giving the stress distribution in the capping skin between the reliefs, but with *d* replaced by *D*, and *x* now measured from the center of that interval region (|*x*|<*D*). Consistent with *σ*_*y*_ being larger than *σ*_*x*_ (Figure 3c) indicates the compressive stress appears to strain concentration and accumulation along the *y*‐direction. When these accumulated compressive stresses are relieved by wrinkling of the capping skin out of the plane, this anisotropic relief of stress produces highly regular patterns perpendicular to the reliefs (Figure 3d).^[^ ^36^, ^95^ ^]^

From what has been discussed above, the prerequisite for the preparation of oriented wrinkles is to first consider the wrinkling system construction, including skin/substrate selection and appropriate force stimulus. While the crucial for the orientation is to introduce anisotropy to the interlaminar stress and guarantee the compressive stress being released anisotropically.

## 4 Fabrication

As depicted above, uniform or equi‐biaxial stresses can result in randomly distributed wrinkles, which constrain the extension of their application. As a result, one of the key challenges in taking advantage of instability‐mediated wrinkling techniques is how to generate periodically patterned surfaces in which long‐range order and controlled distribution of the surface wrinkle patterns is achieved. Therefore, this section will focus on summarizing the main approaches for fabricating oriented wrinkles as a function of different film wrinkling systems and discussing several critical factors considered during the fabrication that can turn wrinkle dimensions as well as orientation in different film wrinkling systems. Finally, a particular focus will be provided on fabricating tunable oriented wrinkles with wavelength and amplitude that can be reversibly modified back and forth.

**Figure 4:**
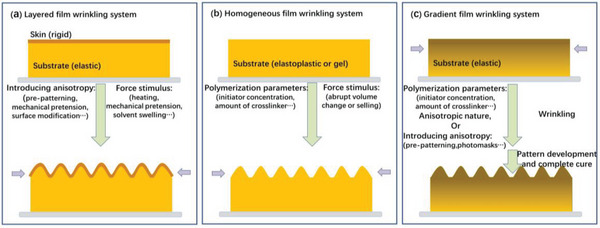
Typical film wrinkling systems capable of fabricating oriented wrinkles: a) Layered film wrinkling system formed by one or several rigid skins capping on an elastic substrate, b) homogenous film wrinkling system composed of homogenously crosslinked hydrogels, and c) gradient film wrinkling system with variable modulus along with the bulk depth.

According to the construction of the precursor film, there are currently three major wrinkling systems employed to fabricate oriented wrinkles: i) layered film wrinkling systems,^[^ ^53^, ^97^, ^98^, ^99^ ^]^ ii) homogenous film wrinkling systems composed of homogenously crosslinked hydrogels,^[^ ^49^, ^100^ ^]^ and iii) gradient film wrinkling systems with variable modulus from the skin surface to the bulk,^[^ ^64^, ^101^, ^102^ ^]^ as illustrated in **Figure** **4**.

### 4.1 Layered Film Wrinkling Systems

Layered film wrinkling systems are composed of one or several rigid skins capping on an elastic substrate, skin/substrate materials should differ significantly in mechanical properties to facilitate wrinkling. To comprehensively depict how oriented wrinkles can be obtained from such systems, we will analyze separately three aspects. First, we will introduce the strategies used to construct a rigid skin. It then considers the type of force stimulus applied to induce the deformation of the skin and the elastic substrate. In the final aspect, we will describe in detail the ways employed to introduce anisotropy for anisotropic wrinkling and the parameters that can provide control over wrinkle dimensions and orientation.

#### 4.1.1 As a Function of Surface Treatment for Constructing Rigid Skin Layers

Skins on the elastic substrate material are currently obtained by employing two main strategies. An elastic substrate (polydimethylsiloxane (PDMS),^[^ ^103^, ^104^ ^]^ shape memory polymers,^[^ ^105^, ^106^ ^]^ polymeric liquid crystal elastomers,^[^ ^107^ ^]^ hydrogels,^[^ ^108^, ^109^ ^]^ and viscoelastic liquid substances^[^ ^110^ ^]^ among others) is either deposited with a thin film of high modulus^[^ ^95^, ^111^ ^]^ or is exposed in surface treatment equipment for altering the modulus of the substrate surface (i.e., direct oxidation on PDMS to generate a stiff silicate‐like skin layer).^[^ ^112^, ^113^ ^]^

##### Deposition of a Thin Film with High Modulus

General polymers (e.g., polystyrene (PS) and polymethyl methacrylate), organic crosslinked polymers (polyaniline, polyvinyl diacrylate, and 2‐hydroxypropyl methacrylate),^[^ ^47^ ^]^ metals (e.g., Al, Au, Ag, Pb, and Ti),^[^ ^114^, ^115^, ^116^ ^]^ inorganic nonmetals (e.g., silicon oxide and aluminum oxide),^[^ ^117^ ^]^ carbon materials (e.g., graphene and carbon nanotubes)^[^ ^37^, ^104^, ^118^ ^]^ as well as self‐assembled multilayer films (e.g., polyallylamine hydrochloride and sodium polystyrene sulfonate)^[^ ^119^, ^120^ ^]^ can all be employed as the skin layers. Depending on the material, spin coating, in situ adsorption polymerization, chemical vapor deposition, sputtering and spraying, film transfer, and layer‐by‐layer self‐assembly methods can be selected for deposition, respectively. The wrinkle dimensions are directly related to the film material applied. According to an overview study carried out by the Fery's group, in general, metal coatings allow sub‐micron wrinkles to be produced, while rigid polymeric coatings allow wrinkles between 2 and 10 µm to be prepared.^[^ ^121^, ^122^ ^]^

##### Direct Surface Treatments

Some polymeric elastic substrates undergo direct oxidation typically using plasma treatment^[^ ^62^, ^123^ ^]^ or UV‐ozone,^[^ ^63^, ^124^, ^125^, ^126^ ^]^ a new skin is created on the substrate surface, thus forming a skin/substrate wrinkling system together with the original substrate. The oxidative method and the operating conditions allow surface wrinkles ranging from a few nanometers to several hundred microns to be obtained, their wavelength can be controlled by varying the duration of the oxidation and predicted according to the following equation^[^ ^123^ ^]^

(1.10) $$\lambda =4.36t{\left[\frac{E_{s}(1-v_{p}^{2})}{E_{p}(1-v_{s}^{2})}\right]}^{1/3}\approx 4.4t{\left(\frac{E_{s}}{E_{p}}\right)}^{1/3}$$

where s and p are the oxidized skin and unoxidized bulk polymer, respectively, and *t* is the thickness of the oxidized skin.

However, the dissimilar mechanical resistance of the skin restricted to specific metals, oxidized skin, or particular high modulus polymers in contrast to the bulk constrains their lifetime and may cause surface cracking. Moreover, the operation processes demand strict experimental conditions (high vacuum systems) or expensive treatment equipment. Equally, these approaches are rather difficult to achieve for large‐area fabrication of wrinkles as well as their long‐term stable application.

**Figure 5:**
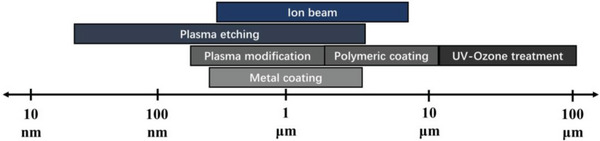
An overview of the wavelength range of wrinkling patterns obtained using various approaches to modify the surface of soft substrates.

Other surface treatments, such as plasma etching (RIE) or ion beam, have been employed to build up skin layers. For instance, when using fluorinated gases to treat PS thin films, controlling the RIE exposure time allows for wavelengths ranging from several micrometers to 30 nm.^[^ ^127^, ^128^ ^]^ Similarly, by varying the fluence and area of exposure, PDMS films can be treated with ion beams to produce nanoscale wrinkling patterns.^[^ ^99^, ^129^ ^]^ As a summary, the wavelength ranges of the wrinkles obtained using various approaches to modify the surface of soft substrates are comprehensively reviewed, as depicted in **Figure** **5**.

#### 4.1.2 As a Function of the Force Stimulus to Induce Surface Wrinkling

There are currently two main force stimulus strategies employed to induce surface wrinkles: those imposed by external forces (mainly heating, mechanical pretension, and solvent swelling) or those imposed by internal forces (mainly provided from within the system).

##### Thermal Response

Thermal response, priority needs to be given to the discrepancy in the thermal coefficient of expansion between the skin and the substrate. Normally, expansion occurs during the heating of the elastic substrate. Substrates with a deposition or physical surface treatment in advance at room temperature or in an expanded state generate a compressive stress in their skin upon heating or cooling, followed by the formation of wrinkles with uniform wavelength.^[^ ^53^, ^106^, ^130^ ^]^ It is worth noting that although some metal films are regularly employed as skin layers, an intermediate layer of titanium or chromium has to be provided to improve poor adhesion, which will complicate the process engineering.^[^ ^86^, ^111^, ^131^ ^]^

##### Mechanical Pretension

Mechanical pretension, emphasis should be paid to the difference in elastic modulus between the skin and the substrate. In general, the skin has an elastic modulus at the GPa level, while the substrate is at the MPa level. Typically, thin‐film deposition or surface treatment on the uniaxially prestrained substrate is carried out to obtain skin. Upon stress release, sequential “wavy” wrinkles are observed. It is the direct result of the modulus mismatch between layers.^[^ ^57^, ^123^, ^132^, ^133^ ^]^

##### Solvent Swelling

Solvent swelling, the difference in swelling coefficient between the skin and the substrate needs to be focused on. In a given solvent atmosphere, the swelling effect and its discrepancy in the penetration of the skin and substrate create compressive stress in the skin, forming regular wrinkles.^[^ ^62^, ^134^, ^135^, ^136^, ^137^ ^]^ Swelling‐induced wrinkling is a reversible process where the solvent controls the disappearance and regeneration of the wrinkles. The rheological properties of the polymer and the geometry of the diffusion front make a crucial contribution to controlling the final wrinkle dimensions and orientation.^[^ ^138^ ^]^

##### Internal Force Stimulus

Internal force stimulus is mainly provided by a crosslinking‐induced volumetric contraction of precursors for elastic substrates (e.g., PDMS).^[^ ^84^, ^86^, ^139^, ^140^ ^]^ Typically, the liquid precursors are first cast onto a stiff skin, subsequently wrinkles on the elastomer substrate are formed after crosslinking‐contraction of the precursors and releasing of water‐soluble polymers as sacrificial layers, where the crosslinking‐induced volumetric contraction of the precursors provides the driving force for developing and stabilizing surface wrinkle.^[^ ^139^ ^]^ This internal force stimulus simplifies the wrinkling procedure since no additional external forces have to be introduced.

#### 4.1.3 As a Function of Applying Anisotropy to Induce Anisotropic Wrinkling

##### Prepatterning

Anisotropic wrinkling can be accomplished by prepatterning the skin or substrate surface of a layered film wrinkling system. In the early days, relief structures or PDMS molds with a certain periodic pattern are often used as physical constraints to control the orientation of the wrinkle.^[^ ^83^, ^94^, ^95^, ^111^, ^141^ ^]^ As a consequence of the confinement of the prepatterned topography, the compressive stress is relieved anisotropically along one direction by wrinkling thus resulting in highly regular oriented wrinkle patterns. Other works are reported to employ photolithography to introduce a series of parallel strips on the capping skin surface.^[^ ^53^, ^142^ ^]^ The interlaminar lateral stresses first occur strain concentrations at the edges of the stripes, and these accumulated lateral stresses result in compressive stresses in the capping skin, generating oriented wrinkles perpendicular to the stripes.

In oriented wrinkles generated over layered film wrinkling systems, the thickness of the capping skin directly influences the dimensions of the wrinkles. According to Equation (1.3), the amplitude increases with increasing thickness, which has been confirmed in widespread studies.^[^ ^81^, ^89^, ^112^, ^123^, ^143^ ^]^ A most interpretative example for analyzing the impact of skin thickness is Qian and co‐workers' investigation. The skin thickness is precisely controlled by employing spin coating, allowing the parameter to be systematically studied. It was found that the wavelength of the wrinkles grew from 3 × 10^−3^ to 7.1 × 10^−3^ nm as the thickness of the PS film increased.^[^ ^81^ ^]^ However, it is worth noting that the flexural rigidity of the soft substrate is greatly dependent on the skin thickness. An excessively thick skin means that the soft substrate requires greater compressive stress to overcome the rising pressure for deformation with increasing skin thickness.^[^ ^81^ ^]^ Instead, if the skin is too thin, the interlaminar lateral stress will be too weak to induce wrinkling.^[^ ^83^ ^]^ Therefore, the thickness of the skin should be rationalized by utilizing the methods for the construction of rigid layers mentioned in Section 4.1.1.

**Figure 6:**
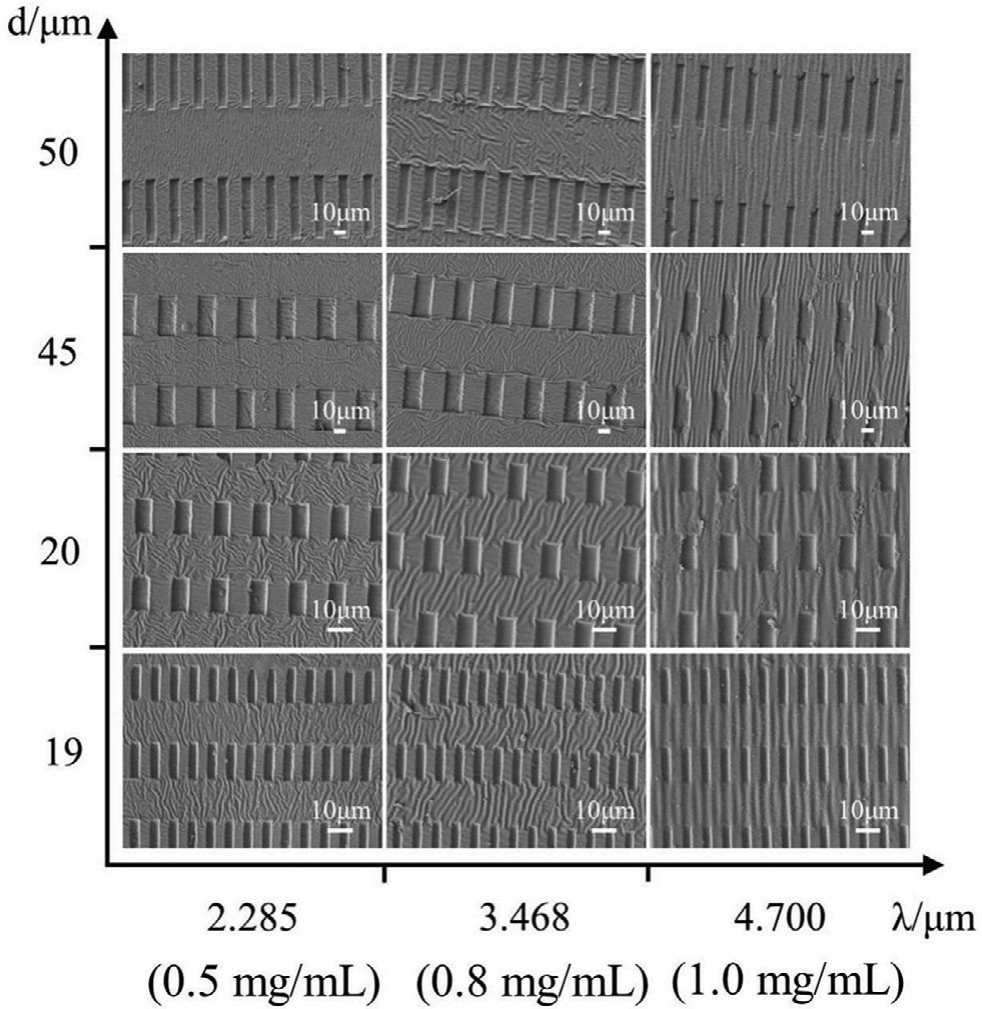
SEM images of grooves‐patterned PDA/PS with different wrinkle periodicity *λ* and distance *d* between adjacent grooves in the long direction. Reproduced with permission.^[^ ^83^ ^]^ Copyright 2017, Elsevier.

In principle, the thickness of the skin is supposed to only have an effect on amplitude. However, not only the wrinkle dimensions but also the wrinkle orientation can be remarkably fine‐tuned.^[^ ^42^, ^83^, ^144^ ^]^ For instance, Wang et al. described polydopamine (PDA)/PS bilayer films that were patterned with microgrooves for forming oriented wrinkles. They constructed PS substrates with grooves of four geometric dimensions and subsequently deposited three different thicknesses of PDA films on each PS substrate. It was found that the orientation of the wrinkles increased with PDA thickness,^[^ ^83^ ^]^ as displayed in **Figure** **6**.

**Figure 7:**
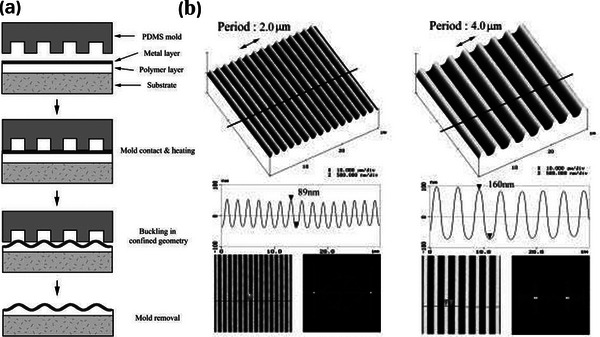
a) Schematic illustration of the procedure employed to produce periodic wrinkling patterns. A PDMS mold with a periodic pattern was placed onto a bilayer metal/polymer film, buckling subsequently takes place when heating above the glass transition temperature of the polymer, finally the mold is removed. b) Ordered wrinkle structures with different wavelengths were obtained at the equal line‐and‐space mold with different periods. Reproduced with permission.^[^ ^94^ ^]^ Copyright 2002, John Wiley & Sons Inc.

The wrinkle dimensions and orientation are also directly related to the period of the applied pattern.^[^ ^53^, ^83^, ^94^, ^142^, ^145^ ^]^ For instance, Yoo's group depicted how the wrinkle dimensions varied as a function of the prepatterned period employed (see **Figure** **7**). They observed that an increase in the period of the equal line‐and‐space mold resulted in an increment in wavelength from 2.6 to 4.2 µm and an increment in amplitude from 89 to 160 nm (see Figure 7b).^[^ ^94^ ^]^ However, the period should be maintained within a suitable range. Yoo's group reported that larger periods cause the resulting oriented pattern to only form in the vicinity of the prepatterned underlying polymer surface such that only local, not global, ordering takes place.^[^ ^36^, ^45^, ^81^, ^83^, ^95^, ^142^ ^]^ A similar phenomenon has been reported by Okayasu et al., they prepatterned an Al/PS bilayer film by a standard photolithographic process to obtain a surface morphology consisting of a series of linear features with varying lateral dimensions. When the topographic stripes were less than 20 µm apart, the surface wrinkles were nearly perfectly parallel. When the gap separation between the stripes increased, the wrinkles became less highly aligned.^[^ ^53^ ^]^

Although the approaches above can fabricate oriented wrinkles without defects over a large area up to several square centimeters, the prepatterning has the limitation of multistep processes or forming discrete and narrow strips, and only tunes wrinkle structures to some extent and lacks the ability to precisely regulate a wrinkled system with arbitrary profiles, particularly in terms of reorganizing wrinkle orientation and position on a 2D surface.^[^ ^36^ ^]^

**Figure 8:**
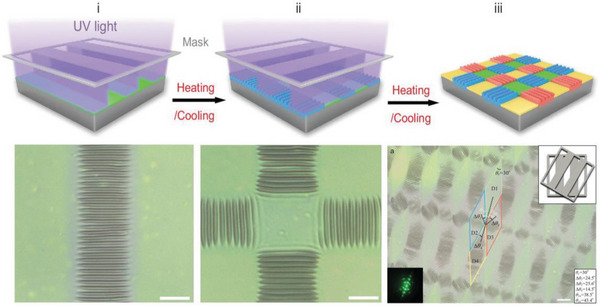
Fabrication of light‐controlled ordered wrinkle patterns by a lithography‐compatible sequential exposure strategy. The corresponding micrographs were fabricated by sequential 365 nm UV light irradiation of a 200 µm strip array photomask. Adapted with permission.^[^ ^36^ ^]^ Copyright 2020, Oxford University Press.

For modulating wrinkle direction and location on demand, Zhou et al. proposed a novel light‐controlled strategy for surface wrinkles. They first prepared a bilayer system by spin‐coating of an anthracene‐contained copolymer on a PDMS substrate, where the modulus of the top photosensitive layer was controlled by UV exposure time. Upon irradiation by UV light with a strip photomask and a further heating treatment, ordered wrinkles were spontaneously generated in the exposed area along single direction because of the mismatch in the modulus and thermal expansion coefficients of the stiff skin and the soft substrate (see **Figure** **8**). By rotating the photomask horizontally at a certain angle, multiple regulation distributions of the ordered wrinkles could be achieved. This novel light control strategy allows dynamic and precise regulation for all the essential features of the wrinkle in the 2D plane, including wavelength, amplitude, direction, and location. More importantly, it establishes a general technical standard for large‐scale fabrication and direction control of 2D ordered wrinkles on demand.^[^ ^36^, ^146^ ^]^

##### Mechanical Pretension

Mechanical pretension allows the wrinkle dimensions and their orientation to be more precisely regulated, therefore this approach is currently one of the most extended and has been widely employed.^[^ ^10^, ^39^, ^89^, ^117^, ^147^, ^148^, ^149^ ^]^

**Figure 9:**
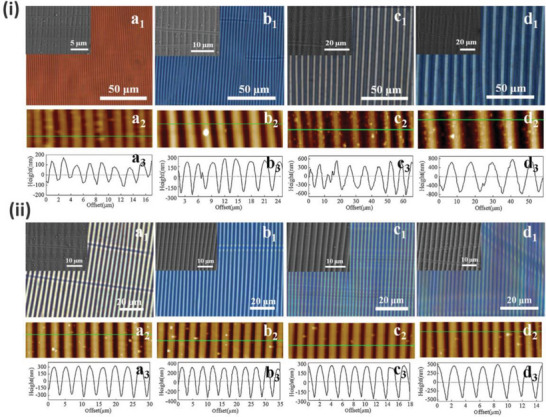
i) a_1_–d_1_) Optical, a_2_–d_2_) AFM height images, and a_3_–d_3_) cross‐section profiles of the PDA/PDMS bilayer from polymerization time: a) 6, b) 12, c) 18, and d) 24 h (d), respectively. ii) a_1_–d_1_) Optical, a_2_–d_2_) AFM height images, and a_3_–d_3_) cross‐section profiles of the PDA/PDMS bilayer from prestrain: a) 10%, b) 20%, c) 30%, and d) 40%, respectively. Reproduced with permission.^[^ ^89^ ^]^ Copyright 2016, Elsevier.

Wrinkle dimensions are directly related to the skin layer thickness and prestrain. A general trend has been observed in which wavelength and amplitude increase with increasing skin thickness; wavelength decreases with increasing prestrain while amplitude increases.^[^ ^89^, ^106^, ^150^, ^151^ ^]^ As an illustrative representative, the role of skin thickness and prestrain in wrinkle dimensions was reported by Meng et al. (see **Figure** **9**). In their work, PDA films were coated on the prestrained PDMS substrates, and striped wrinkle patterns were obtained followed by slow release of the prestrain. In the case of constant prestrain, they observed an increase in the wavelength and amplitude with film thickness (see Figure 9i). In the case of constant film thickness, they carried out a series of prestrain for tests. Consequently, the wavelength decreased with increasing prestrain while the amplitude increased^[^ ^89^ ^]^ (see Figure 9ii). On the downside, a larger strain will yield more cracks and defects.^[^ ^51^, ^89^, ^106^, ^150^, ^151^ ^]^

The intensity of the strain applied not only affects the dimensions but even the type of wrinkle pattern.^[^ ^59^, ^62^, ^152^ ^]^ Some studies have shown that a film can buckle into different patterns ranging from checkerboard, herringbone, hexagonal, triangular, and even labyrinth patterns when subjected to uniaxial/biaxial compressive stresses. The selection and mutual transition of these wrinkling modes hinge on the stress state and the loading history.^[^ ^43^ ^]^ These findings emphasize the significance of force conditions on the fabrication of oriented wrinkle patterns, a critical consideration for applications wishing to exploit the spontaneous formation of periodic patterns caused by surface wrinkling.

Although mechanical pretension has been extensively proven to generate 2D oriented wrinkles, traditionally the dimensions of the substrates used (mostly rectangular) are limited to the centimeter level, and the excessive dimensions make it difficult to apply extensive and uniform stresses. Moreover, the manufacturing process relies on batch processes in a piece‐by‐piece fashion, which limits the continuous manufacture of oriented wrinkles. As a result, the traditional pretension is ineffective in the fabrication of oriented wrinkles.^[^ ^84^, ^147^ ^]^

**Figure 10:**
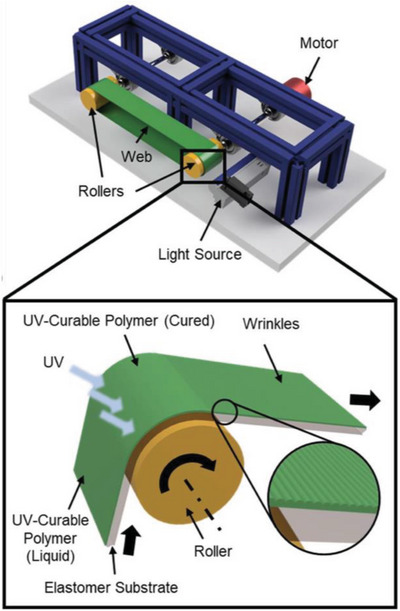
Schematic illustration of the bending‐induced surface wrinkle manufacturing prototype. A liquid UV‐curable polymer is coated on a soft elastomer sheet and is cured by UV light on the curved surface. Reproduced with permission.^[^ ^147^ ^]^ Copyright 2020, American Chemical Society.

To solve this conundrum, Zhang et al. demonstrated a scalable manufacturing method for surface wrinkles in which a cylindrical support was employed to provide bending‐induced strains (**Figure** **10**). Liquid PDMS precursors were spin‐coated onto the surface of a PDMS elastomeric substrate, the resulting bilayer sheet was wrapped onto a cylindrical roller and underwent a bending strain. After UV/ozone treatment, the top liquid film was cured and solidified to act as a stiff layer. During the roller rotating, the bending strain in the PDMS substrate layer was released as the bilayer flattens, thus generating oriented wrinkles. This novel roll‐to‐roll prototype with strain engineering achieved by surface curvature demonstrates a scalable technique for fabricating large‐area oriented wrinkles.^[^ ^147^ ^]^

##### Direct Surface Modification by Ion Beam

Direct ion beam treatment can also be applied to induce anisotropic wrinkling.^[^ ^99^, ^129^ ^]^ Moon's group first reported on the theory that a substrate surface undergoes anisotropic plastic deformation induced by ion beam irradiation.^[^ ^99^ ^]^ Constrained by the elastic substrate, the stiff skin gradually experiences in‐plane compressive strain and tends to expand in the direction perpendicular to the direction of ion beam irradiation. Loading exceeding the critical value results in skin buckling to release the induced mismatch strain between the layers.

The wavelength and amplitude of the wrinkles can be achieved at the micrometer and submicrometer levels through varying the ion beam fluence. Moon's group reported that the dimensions were primarily a function of the ion beam fluence, and increasing the fluence resulted in an increase in the wavelength and amplitude. When the fluence was increased from 1 × 10^13^ to 3 × 10^14^, the wavelength increased from 460 nm to 8.5 µm.^[^ ^99^ ^]^

**Figure 11:**
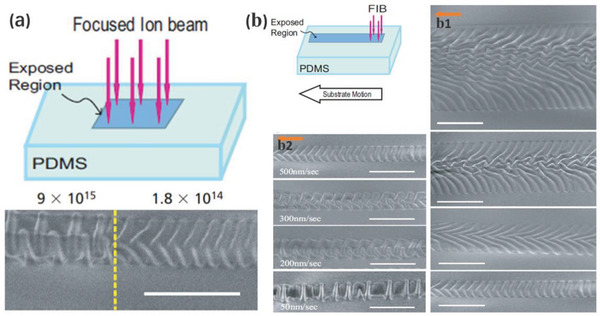
a) Wrinkle patterns on PDMS surface induced by FIB irradiation. b) Wrinkle patterns on PDMS surface induced by controlling the width of the exposed area and the relative motion speed of the polymer substrate. Reproduced with permission.^[^ ^99^ ^]^ Copyright 2007, National Academy of Sciences of the USA.

Wrinkle orientation is primarily a function of the ion beam fluence, the width of the exposed area, and the relative motion speed of the polymer substrate. Moon's group observed that when exposing the substrate to the ion beam irradiation, an increase in the fluence could significantly decrease the wrinkle orientation with a constant width of the exposed area (see **Figure** **11**a). When the substrate was moved at a constant speed relative to the ion beam, the wrinkle orientation was significantly enhanced by reducing the width of the exposed area from 20 to 4 µm (see Figure 11b, _1_). However, when the width of the exposed area was kept constant at 4 µm, the orientation became significantly worse as the substrate was moved at a lower speed (see Figure 11b, _2_).^[^ ^99^ ^]^

The patterning capabilities of this technique are extended by adopting the maskless compared to prepatterning. However, this technique only allows for the creation of controllable wrinkling patterns on small surfaces of polymers. Furthermore, utilizing external irradiation fields attempts to alter some of the mechanical properties of substrate materials to control wrinkled topographies, yet these properties, particularly the modulus and Poisson's ratio, are very difficult to precisely regulate spatially and temporally.

### 4.2 Homogenous Film Wrinkling Systems

Homogenous film wrinkling systems consisting of elastoplastic polymer or crosslinked gel will undergo an abrupt volume phase transition upon swelling with a preferred solvent^[^ ^61^, ^64^, ^153^, ^154^, ^155^ ^]^ or drying from a swollen state,^[^ ^156^, ^157^ ^]^ which induces mechanical instability of the bulk and results in wrinkling.^[^ ^43^, ^154^, ^156^, ^158^, ^159^ ^]^

**Figure 12:**
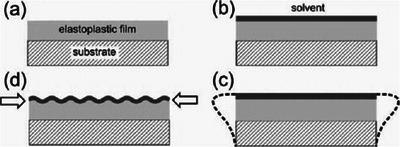
Schematics of swelling‐induced wrinkling process. a) A thin film composed of elastoplastic polymer is coated on a hard substrate; b) the elastoplastic film is swollen by a preferred solvent; c) an abrupt volume phase transition upon swelling; d) resulting in a compressive stress and occurs wrinkling. Reproduced with permission.^[^ ^160^ ^]^ Copyright 2010, John Wiley & Sons Inc.

In the case of swelling‐induced surface patterns, the solvent penetrates into the interior of the film when the polymer film surface is covered with solvent (**Figure** **12**). The bottom of the polymer film is physically constricted by the stiff substrate and only its upper surface can expand, which leads to the generation of anisotropic osmotic pressure along the depth of the film and results in a compressive stress on the film. When the stress is large enough, random wrinkling patterns are usually generated to release the excessive stress and bring the system to minimum energy.^[^ ^159^, ^160^, ^161^, ^162^ ^]^

**Figure 13:**
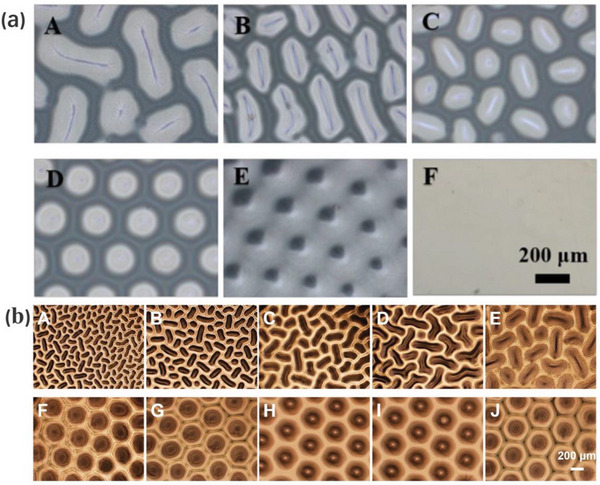
a) Swelling‐induced wrinkling patterns on crosslinked PHEMA films with various amount of crosslinker ethylene glycol dimethylacrylate (EGDMA), where the contents of EGDMA, A‐F, were 0, 2, 4, 6, 8, and 10 wt%, respectively. Reproduced with permission.^[^ ^100^ ^]^ Copyright 2019, American Chemical Society. b) Wrinkling patterns were generated on PHEMA films with different thicknesses. Film thicknesses of A‐J are 160, 240, 320, 400, 480, 560, 640, 720, 800, and 880 µm, respectively. Reproduced with permission.^[^ ^49^ ^]^ Copyright 2021, American Chemical Society.

In oriented wrinkles generated over homogenous film wrinkling systems, the dimensions and orientation of the patterns are directly related to film material, initiator concentration, amount of crosslinker, and film thickness. Many hydrogels, e.g., polyacrylic amide^[^ ^163^ ^]^ or poly(*N*‐isopropylacrylamide) (pNIPAM),^[^ ^164^, ^165^ ^]^ can be swollen to induce wrinkling. However, the resulting patterns are often irregular, which limits their practical application. It has recently been reported that poly(2‐hydroxyethyl methacrylate) (PHEMA) hydrogels can generate a broad range of wrinkling patterns when swelling. More importantly, optimizing the film composition allows the formation of ordered hexagonal patterns.^[^ ^49^, ^100^, ^166^, ^167^ ^]^ This situation has been described by Yun et al. who achieved a smooth PHEMA film surface capable of producing regular honeycomb‐like wrinkling patterns by optimizing the composition of the prepolymer solution.^[^ ^100^ ^]^ When the initiator concentration was in a very narrow range of 0.070–0.074 mol L^−1^, smooth PHEMA films could be obtained. The amount of crosslinker was then increased from 0 to 6 wt%, the wrinkling patterns gradually transformed from peanuts‐like patterns to regular hexagonal patterns, as depicted in **Figure** **13**a. The evolution of the patterns depended on the swelling‐produced osmotic pressure in the films. As the crosslinker content increased, the osmotic pressure in the film induced by swelling approached the critical osmotic pressure for the film to wrinkle, thus resulting in ordered hexagonal wrinkling patterns.

The film thickness may also influence the shape of the wrinkling patterns. Universal studies have revealed that increasing the thickness of the crosslinked film induces an increase in wavelengths and orientation.^[^ ^49^, ^100^, ^166^ ^]^ Typically, Chen et al. observed a significant variation in wrinkling patterns and wavelength with the thickness of the PHEMA film employed (Figure 13b). Curing a thicker film caused a relatively high critical swelling degree for wrinkling. The swelling degree of the films was close to the critical swelling degree.^[^ ^49^ ^]^ Therefore, highly ordered wrinkling patterns were generated. Besides the variation in pattern shapes, the characteristic wavelengths first increased linearly with increasing film thickness, and then gradually converged to a constant value at a certain critical thickness. This was attributed to their large thickness, which caused the top swelling layer to be mechanically constrained by the lower gel layer instead of the substrate.

**Figure 14:**
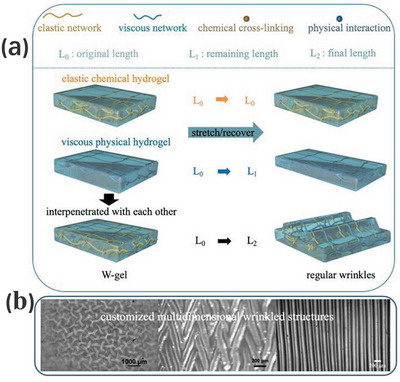
a) Inducing different viscoelastic behaviors inside the double network hydrogel to achieve regular wrinkles after the process of stretch‐recovery. b) Multidimensional wrinkled structures induced by applied stretch in different directions. Reproduced with permission.^[^ ^168^ ^]^ Copyright 2020, Royal Society of Chemistry.

Although various authors have reported the formation of functional oriented wrinkle patterns by controlling crosslinker or initiator concentration,^[^ ^49^, ^100^ ^]^ generally the swelling process imposes large strains on the film, preferring to create creases or even folds. Moreover, the nonlinear feature of the swelling process and inadequate control of osmotic pressure make it difficult to achieve large oriented wrinkled surfaces.^[^ ^45^, ^64^, ^156^, ^168^ ^]^ As an improved alternative in **Figure** **14**, a stretch‐recovery‐induced regular wrinkle formation technique has been applied to double networked hydrogels with distinct viscoelastic behaviors.^[^ ^168^ ^]^ Both compositional networks have different degrees of stretch‐recovery capability. Upon stretching, both networks simultaneously underwent the same extent of deformation, but recovered differently after stress ceases. When interpenetrated together, the contraction force of the viscous gel would induce mass redistribution of the elastic gel, thus forming regular wrinkles (Figure 14a). The pattern dimensions were adjusted by managing the strain intensity and the distribution of the two networks. Depending on the direction of the strains applied, various regular wrinkle structures could be achieved in both 1D and 2D, and they could be well retained in repeated tensile loadings (Figure 14b). Compared with those conventional strategies, this novel approach is much simpler to operate and generates a wider range of wrinkled patterns with better controllability.

### 4.3 Gradient Film Wrinkling Systems

**Figure 15:**
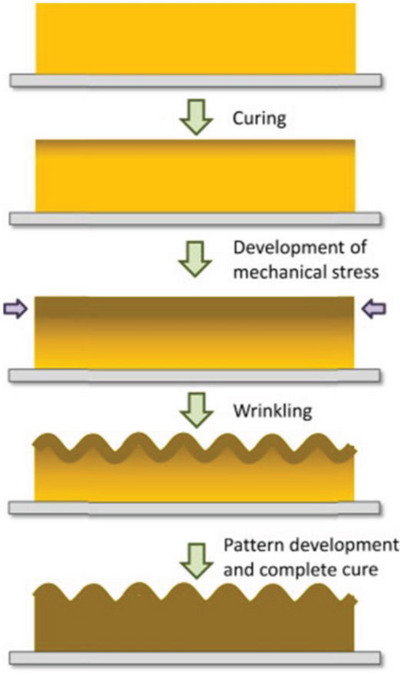
Mechanism for wrinkle formation in a gradient film wrinkling system. The cure is faster at the free surface thus resulting in a rigid layer, followed by the swelling effect as well as local strain produced by further crosslinking of the liquid film bulk driving the development and stabilization of wrinkling morphology. Reproduced with permission.^[^ ^45^ ^]^ Copyright 2015, Elsevier.

Films with a modulus gradient will occur wrinkling upon solvent swelling. Basu's group was first to provide a well‐accepted mechanism for wrinkle formation in such a system,^[^ ^169^ ^]^ as depicted in **Figure** **15**. Upon heat or/and UV irradiation, a homogenously crosslinked liquid film is gradually cured and simultaneously develops a gradient in degree of crosslinking and mechanical properties as the film depth. A larger crosslinking extent of the liquid film surface layer can act as a rigid skin, followed by the swelling effect as well as local strain produced by further crosslinking of the liquid film bulk driving the development and stabilization of wrinkling morphology.^[^ ^64^, ^154^, ^160^ ^]^ The resulting wrinkles are also usually randomly distributed, thus different alternatives can be employed to control wrinkle dimensions and orientation.

#### 4.3.1 Polymerization Parameters

As an alternative to the use of homogenously crosslinked films, equally, the experimental conditions chosen for gradient crosslinked films will determine the basic characteristics of the resulting wrinkles. Among the parameters that can be modified, we can remark film material, initiator and crosslinker concentration, and film thickness.^[^ ^64^, ^102^ ^]^

**Figure 16:**
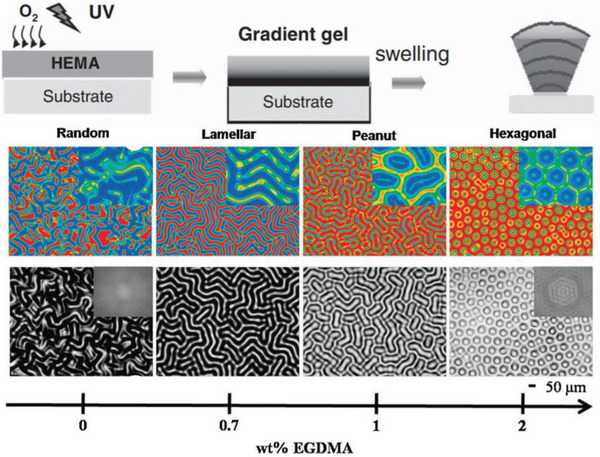
Schematic illustration (above) and optical images (below) of patterns spontaneously formed for PHEMA hydrogel films with a modulus gradient upon swelling, where the order of the patterns is determined by the concentration of crosslinker. Reproduced with permission.^[^ ^160^ ^]^ Copyright 2010, John Wiley & Sons Inc.

In this concern, an illustrative work was reported by Guvendiren et al., they revealed on the dynamic evolution of extensive patterns on the surface of PHEMA hydrogel gradient crosslinked films (**Figure** **16**). For films thicker than 20 µm, the amount of oxygen diffusing into the film gradually decreased, thus manipulating the UV curing process to prepare films with progressively increasing crosslink density. The local osmotic pressure within the film could be modulated by the amount of initiator, crosslinker concentration, exposure time, and intensity, with highly ordered hexagonal patterns forming as the crosslinker concentration increased to 2 wt%. The characteristic wavelengths of the patterns increased linearly with film thickness and decreased with increasing crosslinker concentration. The pattern orientation, however, depended on the crosslinker concentration and was independent of the film thickness.^[^ ^64^ ^]^

#### 4.3.2 Utilizing the Anisotropic Nature of Liquid Crystalline Polymer (LCP)

Anisotropic wrinkling exploiting the anisotropic nature of LCP has been found to provide another alternative of physical self‐assembly to fabricate periodic microstructures.^[^ ^170^, ^171^, ^172^, ^173^ ^]^ LCP, which incorporates anisotropic mesogenic groups into polymer chains, is a polymer having liquid crystal (LC) state in molecular ordering, and it exhibits both high mobility and excellent anisotropic characteristics in the LC state. As a result of its anisotropic nature, it has been used in the microfabrication of oriented wrinkled surfaces.^[^ ^101^, ^174^ ^]^

**Figure 17:**
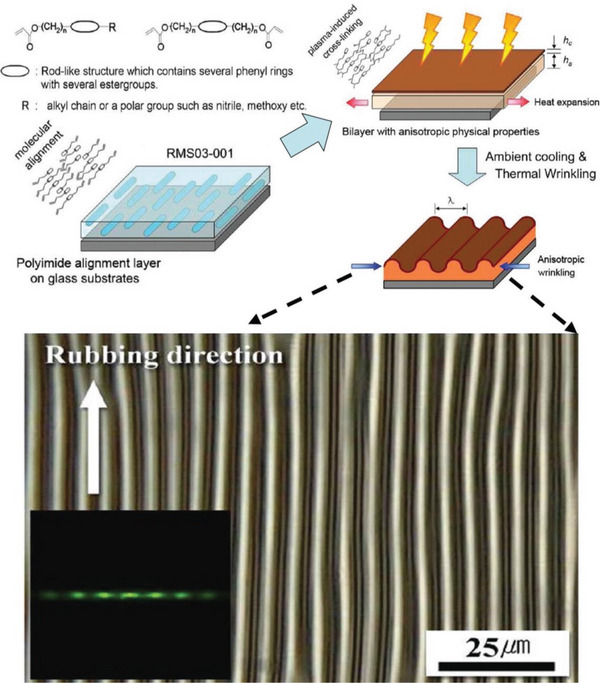
Schematic illustration of the anisotropic wrinkling process which originated from the molecular orientation of LCP, where plasma treatment on the aligned RMS film constitutes the bilayer system of the crosslinked top skin layer on the nonpolymerized RMS solution. 1D wrinkle pattern formed by utilizing anisotropic nature of an aligned LCP. Reproduced with permission.^[^ ^101^ ^]^ Copyright 2012, American Chemical Society.

In this concern, an illustrative work was reported by Kang et al., see **Figure** **17**.  They spin‐coated a reactive mesocrystalline solution (RMS) onto a polyimide (PI) film rubbed by a roller‐type rubbing machine, where the RMS molecules were aligned along the rubbing direction. During plasma exposure, the RMS films of the aligned LCP expanded and generated compressive stresses. When the accumulated compressive stress exceeded a critical value, wave patterns spontaneously formed on the RMS film surface. The wrinkle orientation was tuned by the structural ordering of the LCP molecules and the PI film substrate. They observed that unrubbed PI films led to isotropic wrinkle patterns with discrete random domains. The dimensions of the patterns were directly related to the plasma exposure time and film thickness. The amplitude increased with time in the 60–100 s range. At a constant exposure time, the reduced film thickness led to patterns with shorter wavelengths and smaller amplitudes.^[^ ^101^ ^]^

This nonlithographic technique utilizes the anisotropic nature of the wrinkling system itself without external skin film deposition, prestrain control of the system, or physical confinements. Therefore, it is prospective in large‐area patterning manufacture or roll‐to‐roll processing. However, it has to be mentioned that this approach demands a rather high anisotropic nature of the polymer materials, which may limit the selection of wrinkling materials and its scalability to some extent.

#### 4.3.3 Applying Anisotropy

Besides the above methods, a final alternative is to consider applying anisotropy on the skin surface as applied in layered film wrinkling systems, where prepatterning or applying extra photomasks is typically employed.^[^ ^102^, ^175^, ^176^, ^177^, ^178^ ^]^

In the case of prepatterning, equally, the film thickness and the period of the pattern applied play an important role in the dimensions and orientation of the wrinkles obtained, respectively.^[^ ^176^, ^178^ ^]^ Li et al. reported a method for creating submicrometer oriented wrinkle patterns via reactive silane injection into prepatterned PHEMA film. Continuous injection and condensation of methyltrichlorosilane provided further crosslinking, driving the development and immobilization of the wrinkled structure. Self‐alignment of the wrinkled morphology was directed by thermal nanoimprint lithography and e‐beam lithography on the PHEMA layer before infusion, whereby the obtained wrinkles revealed a preferential alignment perpendicular to the pattern boundary. This ordering arises due to the preferential release of compressive stress perpendicular to the patterned lines during the wrinkling process.^[^ ^95^, ^178^ ^]^ It was possible to control the wrinkle dimensions from the nanoscale to the microscale by simply varying the original film thickness, and the same linear increase trend in dimensions could be observed with an increase in thickness.^[^ ^178^ ^]^ Similar results were also reported by Chen et al. Furthermore, they probed the geometric effect of prepatterned pitch on the wrinkle orientation, see Figure 20. With a gradual reduction in pitch to 20 µm, the wrinkles completely lose their isotropic nature, and their formation is dominated by the edge effect of the prepatterns. As a result, they were aligned perpendicularly to the prepattern in an out‐of‐phase fashion.^[^ ^176^ ^]^

**Figure 18:**
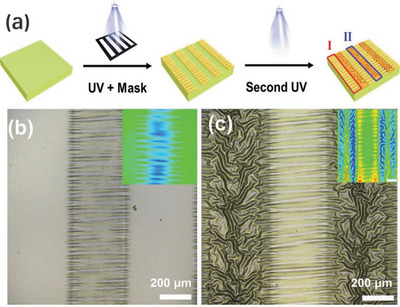
a) The fabricated strategy of strip‐type hierarchical wrinkle patterns; b,c) laser scanning confocal microscopy images of ordered wrinkles and hierarchical patterns after first and second exposure, respectively, where inset pictures are corresponding 1D and 2D morphology images. Reproduced with permission.^[^ ^177^ ^]^ Copyright 2021, John Wiley & Sons Inc.

Even with different film wrinkling systems, restrictions of prepatterning, such as multistep processes, discrete and narrow strips, and the absence of precise regulation for morphology, still exist. Recently, this concern has been addressed by Jiang's group. They demonstrated a simple and robust method for fabrication of the self‐organized ordered patterns on a photosensitive polymer film with gradient mechanical properties, with programmable exposure of the mask controlling the orientation of the wrinkles (**Figure** **18**). They drop‐coated a solution mixture of monomers containing photoinitiators onto the substrate, and the photoinitiators self‐assembled at the gas–liquid interface to form the top layer due to the low surface energy. Under UV light irradiation, the mismatch of shrinkage caused by the gradient photocrosslinking led to the formation of wrinkles. The content of the photoinitiator, the film thickness, and the mass ratio of the monomer could provide control over the dimensions of the wrinkles. Increases in content or thickness could observe an increase in wavelength and amplitude. This novel strategy provides a promising candidate for precise control over the main characteristics of the wrinkles and the large‐scale fabrication of oriented and layered wrinkling patterns with stable topography.^[^ ^177^ ^]^

Films with modulus gradients are comparatively convenient to fabricate in large areas by means of thermal and/or UV curing as well as the obvious physical gradient characteristics. Therefore, in contrast to homogenous film wrinkling systems, solvent swelling of films with modulus gradients is capable of improving the control of film structure on a larger scale, more easily generating oriented wrinkle patterns ranging from parallel lateral, and regular polygons, among others.

### 4.4 Tunable Oriented Wrinkled Surfaces

The enthusiasm for developing tunable or stimulus‐responsive materials springs from their capacity to perform macroscopic variation upon external stimulus.^[^ ^45^, ^179^, ^180^ ^]^ These materials have displayed widespread applications, including switchable wetting,^[^ ^181^, ^182^ ^]^ adhesion control,^[^ ^183^, ^184^ ^]^ optical memory materials,^[^ ^185^ ^]^ smart windows,^[^ ^186^ ^]^ or cellular interactions.^[^ ^187^, ^188^ ^]^ Therefore, it is pretty attractive to create ideal responsive wrinkling systems that are capable of responding to external stimulus in a controlled and predictable manner.

**Figure 19:**
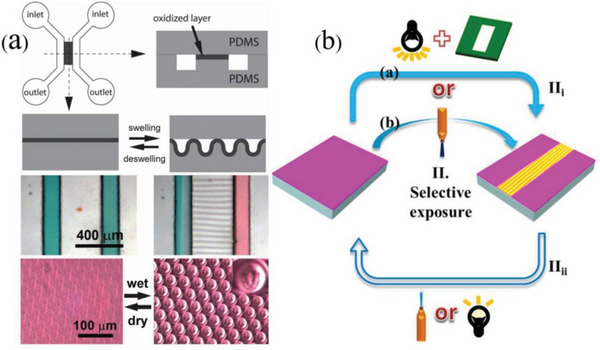
a) Reversible wrinkle channels in a microfluidic device (above); reversible microlens array formed by ethanol‐wetting (below). Reproduced with permission.^[^ ^62^ ^]^ Copyright 2011, John Wiley & Sons Inc. b) Uniaxially oriented wrinkles are induced by exposing the PDO_3_/PDMS bilayer to a high‐intensity white light with a mask (II_i_‐a) or a high‐intensity moving laser beam (II_i_‐b), respectively; optical erasure of surface wrinkles by exposure to a low‐intensity white light or laser beam (II_ii_). Reproduced with permission.^[^ ^190^ ^]^ Copyright 2020, American Chemical Society.

Responsive wrinkled materials can respond to a variety of stimulus ranging from initially mechanical or solvent to recently reported light, gases, pH, humidity, or temperature. Earlier, oriented wrinkled surfaces are typically fabricated with prestrained elastic substrates modified by surface treatment, thereby offering a unique foundation for possessing both reversible and responsive behavior. These functional surfaces with controlled amplitude and periodicity have been applied in modifying surface wettability,^[^ ^39^, ^136^, ^148^ ^]^ dynamic optical switches,^[^ ^189^ ^]^ smart windows,^[^ ^186^ ^]^ among others. Similarly, specific solvents may induce the generation of oriented wrinkles, which disappear when the solvent evaporates. Kim and Crosby have employed this strategy to fabricate permeation‐driven oriented wrinkles,^[^ ^62^ ^]^ as shown in **Figure** **19**a.

Oriented wrinkled surfaces with responsive behavior toward environmental variations, such as light, gases, pH, humidity, or temperature, can either be fabricated by photosensitive or hydrogel materials.^[^ ^165^, ^189^, ^190^, ^191^, ^192^, ^193^ ^]^ The photoresponsive capability of azobenzene‐containing polymers (azopolymers) can be served as building blocks for soft photonics in emerging smart surfaces. Wang et al. reported a visible‐light illumination strategy to trigger ordered orientation of wrinkles on the surface of azobenzene polymers, where the different photoresponsive properties of azobenzene served as the driving force for the wrinkling/dewrinkling switch, switching between flat and wrinkled states by controlling the intensity of the incident light,^[^ ^190^ ^]^ see Figure 19b. In the case of hydrogel materials, Kim et al. recently reported on a design strategy for reversibly forming highly homogenous crease patterns on hydrogel films allowing the ability to control colloidal particles in response to pH changes.^[^ ^192^ ^]^

## 5 Applications of Oriented Wrinkled Interfaces

As depicted above, the oriented wrinkle dimensions, their highly periodic distribution at the surface and convenient for modifying the chemical composition are among the parameters that can be accurately modulated within their fabrication. For that reason, this versatile platform has been employed and is currently being assessed for more potential applications. Herein, the major fields in which the functional properties (wavelength, amplitude, and orientation) of these unique platforms allowed them to be applied in specific examples in recent years are summarized.

### 5.1 As Templates for Constructing Surface Arrays

**Figure 20:**
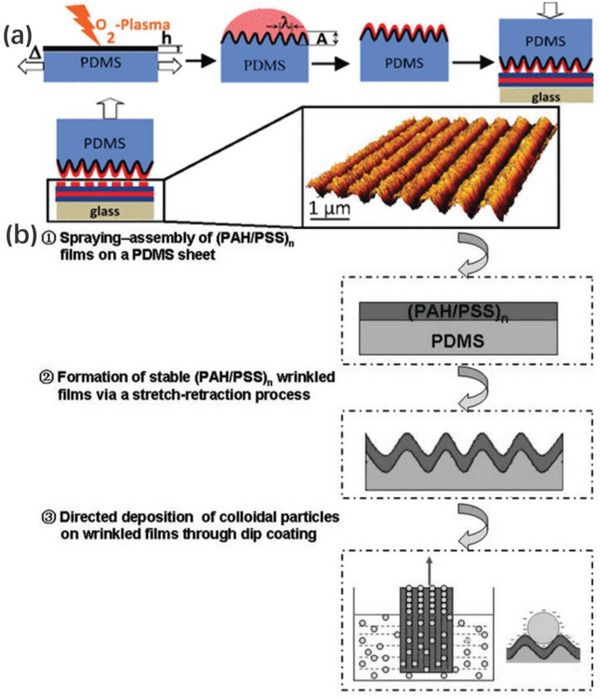
a) Scheme of wrinkle‐assisted microcontact printing. Reproduced with permission.^[^ ^194^ ^]^ Copyright 2008, American Chemical Society. b) Schematic illustration of a three‐step procedure for template‐directed deposition of colloidal particles on wrinkled films. Reproduced with permission.^[^ ^195^ ^]^ Copyright 2007, Royal Society of Chemistry.

Regularly arranged surface array materials play an important role in various fields and are often constructed by expensive photolithography. Oriented wrinkled interfaces with controlled dimensions and orientation can be used as templates to produce ordered patterns in combination with techniques such as microcontact printing. Pretzl team successfully printed patterned macromolecules such as polyelectrolytes and proteins on flat surfaces using wrinkle‐assisted microcontact printing,^[^ ^194^ ^]^ see **Figure** **20**a.

Such interfaces can also be used as templates for the preparation of highly ordered surface arrays in combination with techniques such as microcontact embossing and dip coating methods.^[^ ^38^, ^194^, ^195^ ^]^ Many materials all can be applied to construct surface‐ordered arrays via oriented wrinkled templates. Solid particles such as polymeric microspheres and micro‐nanoparticles, metal nanoparticles and nanowires, and bio‐nanoparticles can be selectively enriched in these wrinkled grooves.^[^ ^195^, ^196^, ^197^ ^]^ Figure 20b illustrates a versatile dip coating strategy for organizing nanoparticles by employing oriented wrinkled surfaces as templates to form controlled ordered arrays, where particles can be selectively enriched in the grooves by electrostatic or capillary forces.^[^ ^195^ ^]^

**Figure 21:**
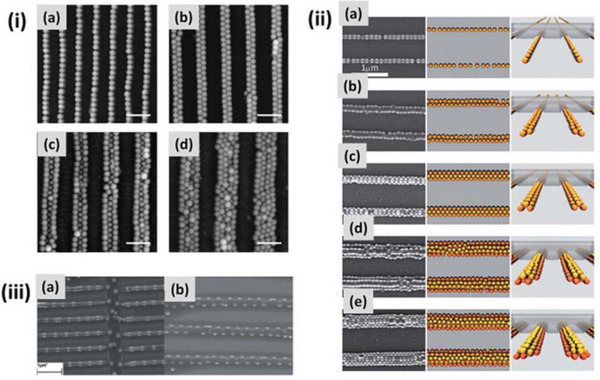
i) AFM images of selective deposition of colloidal particles on wrinkled films with diverse periodicities. ii) Sequence of structures with different surface features formed by modulating the concentration of the colloidal solution from (a) to (e), structures that range from single‐file wires to prisms with triangular cross‐section.iii) Ordered arrays formed using PDMS templates with two different wavelengths (660 nm for A and C; 1200 nm for B and D). Reproduced with permission.^[^ ^45^ ^]^ Copyright 2014, Elsevier.

The above strategy allows for fine tuning of the obtained nanoparticle patterns following the preparation conditions, as depicted in **Figure** **21**. Exemplarily, periodic adjustments allow to change the number of particle lines (i)^[^ ^195^ ^]^ and their distribution within the lines (iii).^[^ ^198^ ^]^ Besides, modulating the concentration of the colloidal solution used allows to obtain a sequence of structures with different surface features. As the concentration increases, specific arrangements of particles varying between single‐line and prismatic patterns are observed (ii).^[^ ^199^ ^]^

### 5.2 Surface‐Enhanced Raman Spectroscopy (SERS) Substrates

Surface‐enhanced Raman scattering (SERS) technology provides ultrahigh sensitivity and outstanding stability for chemical detection and analysis, becoming a promising and powerful spectroscopic tool for the detection of biological and chemical substances in the biopharmaceutical and chemical industries.^[^ ^200^, ^201^, ^202^ ^]^ However, the expensive and complex fabrication of SERS substrates, such as wet and dry etching, limits their practical applications.^[^ ^201^, ^203^, ^204^, ^205^, ^206^ ^]^ Therefore, achieving low‐cost fabrication of rewritable SERS substrates remains a great challenge.^[^ ^207^, ^208^ ^]^

**Figure 22:**
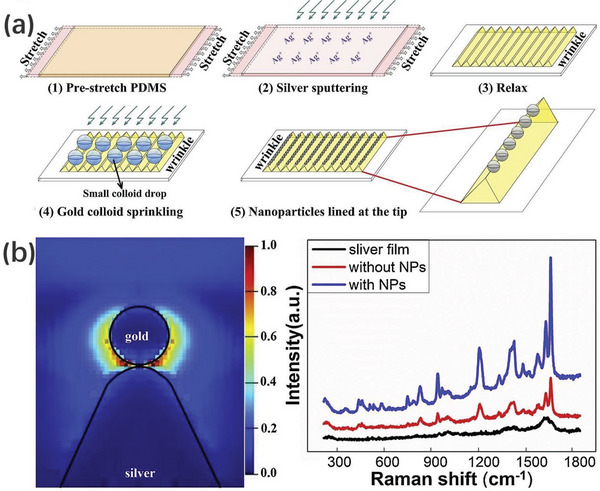
a) Schematic diagram for the fabrication of ordered gold nanoparticle arrays. Silver wrinkled structures were fabricated by sputtering silver nanofilms onto a prestrained substrate. Then, gold nanoparticles were added in line at the top of the structures by dropping a gold colloidal solution. b) SERS effect was enhanced as a result of the hot spot structures between the nanoparticles as well as the wrinkled structures (colored in red). Reproduced with permission.^[^ ^200^ ^]^ Copyright 2019, Elsevier.

Research demonstrates that highly ordered arrays formed by self‐assembly of nanoparticles induced by oriented wrinkled templates are well suited for the fabrication of rewritable SERS substrates. Earlier approaches were carried out by organizing gold nanoparticles on wrinkled surfaces. A typical example, professor Fery and his group induced ordered arrays of optically active gold nanostars coated with pNIPAM in oriented wrinkled templates, where the pNIPAM shells acted both as separation layers to inhibit plasma coupling of the metal cores and as molecular traps to effectively trap hydrophobic analytes. Testing the analytical reagent pyrene with its capability for gas phase detection indicated that this ordered array of AuNS@pNIPAM particles induced by the oriented wrinkled templates showed a significantly enhanced Raman signal.^[^ ^209^ ^]^ Another alternative is available through producing gold/silver films containing SERS‐active nanogaps and sharp nanotip. Guo et al. fabricated SERS substrates with oriented silver winkled structures by sputtering silver nanofilms onto a prestrained PDMS substrate, gold nanoparticles were then added in line at the top of the silver wrinkled structures (**Figure** **22**a). This platform showed a strong Raman enhancement as a result of the hot spot structures between the nanoparticles as well as the silver wrinkled structures (Figure 22b). A detection limit of 10^−20^ m for Cresyl‐Violet and Rhodamine 6 G molecules demonstrated its potential to detect single molecules. Their reported method provides an extremely sensitive SERS substrate for the detection of biomolecules.^[^ ^200^ ^]^

### 5.3 Flexible Electronics

Flexible and stretchable electronics represent the cutting edge of electronics technology today. However, stretchability is generally more challenging to achieve than flexibility. Stretchability demands electronic devices to be able to absorb strain much greater than 1% without significant degradation in their electrical performance.^[^ ^210^, ^211^, ^212^, ^213^, ^214^ ^]^ Conventional conductive materials for electrodes, such as single‐crystal inorganic semiconductors (typically Si),^[^ ^215^, ^216^ ^]^ metals (mainly including Au, Ag, or Al),^[^ ^216^, ^217^ ^]^ or metal oxides (mainly indium tin oxide),^[^ ^218^, ^219^, ^220^ ^]^ have the inherent disadvantage of being brittle and prone to fracture when subjected to external forces. While flexible substrates based on oriented wrinkled surfaces are typically designed and manufactured to provide stretchable functionality without significant degradation in performance.

By spin‐coating conductive, light‐emitting and energy‐storing materials on a prestrained flexible film substrate, flexible electrodes,^[^ ^221^, ^222^, ^223^, ^224^ ^]^ sensors,^[^ ^225^, ^226^ ^]^ and light‐emitting diodes^[^ ^227^, ^228^ ^]^ can be prepared with oriented wrinkled surfaces. This configuration can be reversibly stretched or compressed with an elastic response to strain by adjusting its wavelength and amplitude, involving substantial strains in the elastic polymer substrates instead of the conductive materials themselves. This design is almost identical to an accordion bellow and exhibits significant stretchability.^[^ ^229^, ^230^ ^]^

**Figure 23:**
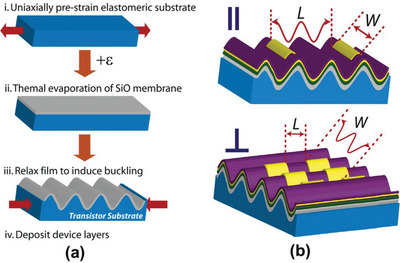
a) Fabrication of organic thin‐film transistors with wavy features by conformal coating methods. b) The two device configurations evaluated in this study are parallel and perpendicular. Reproduced with permission.^[^ ^230^ ^]^ Copyright 2013, Elsevier.

This design exhibits a certain extent of extraordinary flexibility and stretchability. However, a key aspect that remains unresolved is the fracture observed in organic semiconductor films following the release of prestrain to create the wave structure.^[^ ^231^, ^232^ ^]^ As an improved alternative, see **Figure** **23**, Wu's group reported the fabrication of organic thin‐film transistors onto prefabricated wrinkled substrates by conformal coating methods as an alternative to conventional approaches in which coatings were imposed in prestrained films. As a result, this approach could protect multilayer devices from damage resulting from mechanical stress at the same time maintaining high stretchability.^[^ ^230^ ^]^

### 5.4 Controlled Wettability

Controlled surface wettability has attracted great interest in many fundamental and applied sciences. In particular, superhydrophobic surfaces (water contact angle above 150° and rolling angle below 10°) are extremely attractive and are widely applied in areas such as self‐cleaning, anti‐icing, membrane distillation, and oil–water separation.^[^ ^233^, ^234^, ^235^, ^236^, ^237^ ^]^ Wettability is highly dependent on material surface energy and surface roughness, and the introduction of micro‐ and nanohierarchical structures on low surface energy materials is currently the main means of preparing superhydrophobic surfaces, where the hierarchical structures can trap more air and thus prevent the surface from being wetted.^[^ ^238^, ^239^, ^240^, ^241^, ^242^, ^243^ ^]^

**Figure 24:**
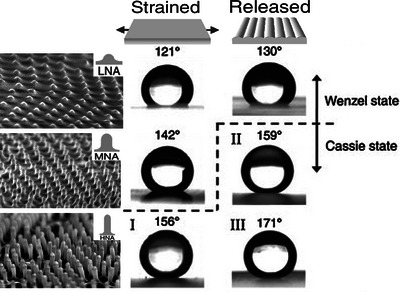
Comparison of water contact angles on the differently structured PDMS surfaces, where hydrophobicity depends on the pillar aspect ratio and the mechanical strain. Adapted with permission.^[^ ^39^ ^]^ Copyright 2010, John Wiley & Sons Inc.

Oriented wrinkled structures with controlled surface functionality and variable roughness can effectively control the wettability of the surface.^[^ ^39^, ^148^, ^244^, ^245^, ^246^, ^247^ ^]^ Lee et al. presented an elastomeric smart window with switchable wetting, as depicted in **Figure** **24**. The nanopillar structure was first replicated onto a PDMS film surface by micromolding. Upon subsequent uniaxial stretch and exposure to UV‐ozone radiation, hierarchical structures of periodically micron‐scale wrinkles and nanopillar arrays were generated on the PDMS surface after releasing the strain. The hydrophobic properties of the surface could be significantly modulated by changing the aspect ratio of the nanopillars or the mechanical strain applied.^[^ ^39^ ^]^ They observed that the contact angle increased when the aspect ratios of the nanopillars increased or microwrinkles were present, which is attributed to the fact that more air is trapped in the microstructure.

In contrast to the previous case, superhydrophilic wetting can also be achieved when high surface energy hydrophilic materials are combined with oriented wrinkled surfaces.^[^ ^248^ ^]^ This is attributed to the wrinkled structure and high surface energy of the composite surface effectively increasing its affinity with water.^[^ ^249^, ^250^ ^]^

### 5.5 Tunable Optical Properties

Oriented wrinkled surfaces are used quite extensively in optical applications, especially in switching the optical properties of specific films, such as microlens arrays,^[^ ^251^, ^252^ ^]^ optical gratings with switchable transparency,^[^ ^39^, ^56^ ^]^ and tunable structural color platform for smart display,^[^ ^206^ ^]^ among others.

**Figure 25:**
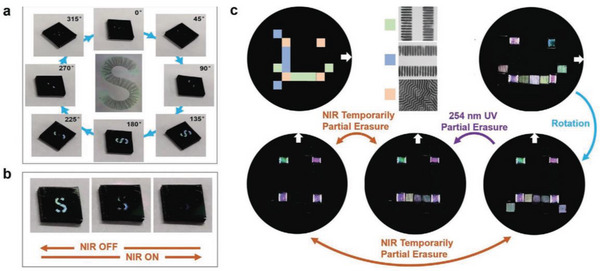
Dynamic structural colors on WTFs. a) Macrographs of structural colors on ordered wrinkle sample under different horizontal *γ* detecting angles. b) Macrographs of dynamic structural colors regulation on ordered WTFs via NIR light switching on and off cycles. c) Macrographs and schematic diagram of structural colors on disordered and ordered WTFs by adjustable horizontal detecting angle or UV/NIR light. Reproduced with permission.^[^ ^206^ ^]^ Copyright 2020, John Wiley & Sons Inc.

A variety of stimulus ranging from initially mechanical or solvent^[^ ^39^, ^253^, ^254^ ^]^ to recently reported light, pH, humidity, or temperature^[^ ^186^, ^206^, ^255^, ^256^ ^]^ can respond to tunability. Lee et al. reported an elastomeric smart window with an optical switch and evaluated its switching capabilities. Extensive scattering of light by the periodic micrometer‐sized wrinkled structures appeared to result in the opacity of the film. When the sheet was extended to its initial stretched length, it gradually became transparent due to the absence of wrinkles and provided a clear transmitted image.^[^ ^39^ ^]^ Recently there has been great interest in taking advantage of surface wrinkling to create reversible patterns that are responsive to external stimuli. Combining spontaneous film interference and wrinkling phenomena, Zhou et al. recently prepared anisotropic structural colors with different optical properties on ordered wrinkled thin films (WTFs), where the dynamic structural colors can be precisely and reversibly regulated by UV light and near‐infrared (NIR) light irradiation (**Figure** **25**).^[^ ^206^ ^]^

### 5.6 Tunable Adhesion/Friction

The fabrication of dry adhesives with inspiration from the outstanding adhesive lubricant interfaces found in nature has attracted global attention. However, the manufacturing approaches that typically employ high aspect ratio structures require specific equipment and time‐consuming processes, which limit their practical application.^[^ ^139^, ^257^, ^258^, ^259^ ^]^ This creates an ideal platform for the application in which oriented wrinkled interfaces with accurately controllable structure and chemistry in enhancing adhesion and friction. Studies have revealed that the adhesion depends on the existence or otherwise of wrinkled areas as well as their special dimensions.^[^ ^153^, ^260^ ^]^ These offer the possibility of designing more sophisticated adhesives.

First, since the adjustable mechanical strain applied allows for reversible modification in the dimensions of the oriented wrinkles, thus making it possible to design mechanically adjustable adhesives.^[^ ^260^, ^261^ ^]^ Furthermore, based on the potential of combining wrinkled and planar areas, the substrate will exhibit regions of high and low adhesion.^[^ ^262^ ^]^ Last but not least, most of the studies reported so far to analyze the adhesive properties have been carried out using wrinkled/planar interfaces. However, an interesting behavior has been found, when using complementary oriented surfaces, i.e., two surfaces with similar wrinkle characteristics make contact, interfacial adhesion shows significant enhancement with increasing ripple amplitude. In contrast, interfaces with mismatched amplitudes had nearly negligible adhesion.^[^ ^263^ ^]^

### 5.7 Enhanced Cell Alignment

In the field of biomaterials science, textured surfaces with periodic topographical features and long‐range order are highly attractive for directing cell–material interactions. They mimic physiological environments more accurately than planar surfaces and can fundamentally alter cell growth, alignment, differentiation, morphology, and cell death.^[^ ^57^, ^86^, ^264^, ^265^, ^266^, ^267^, ^268^ ^]^  Extensive published evidence suggests that aligned surface morphologies are essential for cell alignment, tissue morphogenesis, and tissue remodeling, which provide effective and accurate expression of tissue functions.

Existing techniques capable of creating tunable multiscale features for cell adhesion and alignment, such as laser ablation,^[^ ^269^ ^]^ chemical vapor deposition,^[^ ^270^, ^271^ ^]^ nanoimprint lithography,^[^ ^272^, ^273^ ^]^ nanocasting,^[^ ^274^ ^]^ and chemical etching,^[^ ^275^ ^]^ have drawbacks in expensive equipment, specific high vacuum conditions, time‐consuming and complicated steps (e.g., lithography), and so on. While surface wrinkling technology can offer an elegant alternative to direct molding of surface patterns.

**Figure 26:**
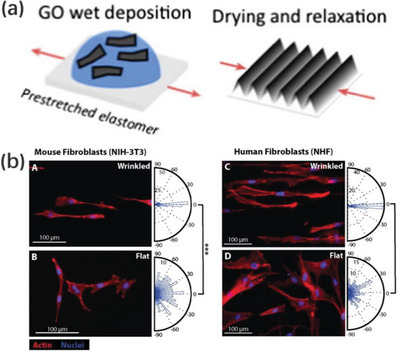
a) Fabrication of wrinkled graphene‐based multilayer films for high alignment of cells. b) Cells appeared highly aligned on the wrinkled substrates (A and C). Reproduced with permission.^[^ ^264^ ^]^ Copyright 2015, Elsevier.

Oriented wrinkled surfaces can enhance cell alignment and stimulate cell adhesion, motility, or proliferation through topographical interactions.^[^ ^264^, ^267^, ^276^, ^277^ ^]^ Wang et al. fabricated periodic, delaminated buckle textures by graphene oxide wet deposition onto prestretched elastomers, followed by relaxation and mild thermal treatment to stabilize the films in cell culture medium. Human and murine fibroblasts attach to these textured films and remain viable, while developing pronounced alignment and elongation relative to those on planar graphene controls (**Figure** **26**b).^[^ ^264^ ^]^

### 5.8 Microfluidic Devices

Microfluidic devices play a paramount role in controlling microsphere screening fluid flow. However, valves regulated by responsive materials (typically including hydrogel,^[^ ^278^ ^]^ thermoreversible gelation polymers,^[^ ^279^ ^]^ or shape‐memory polymers^[^ ^280^ ^]^) are associated with long response times. While the use of external electromagnetic devices often results in complex devices.^[^ ^281^ ^]^ Therefore, it remains a challenge to design and fabricate integrated microfluidic systems with active controls.

**Figure 27:**
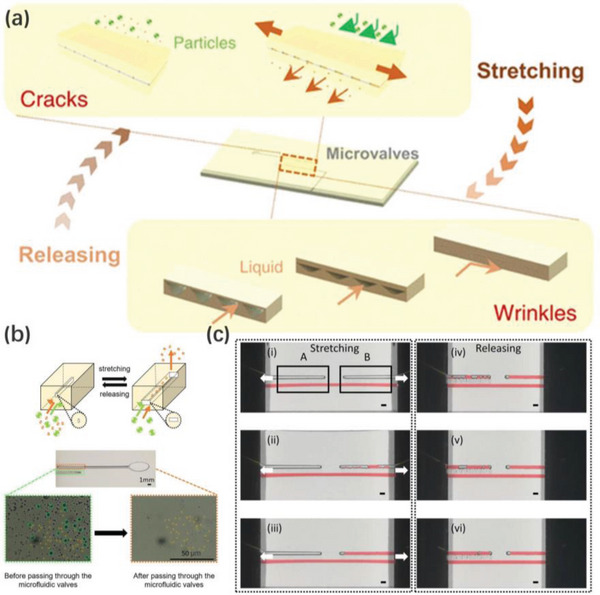
a) The microfluidic devices with strain‐tunable crack and wrinkle microvalves for microsphere screening and programmable microfluidic logic devices. b) Strain‐tunable microvalves for microsphere screening in polydisperse mixture. c) Optical images of the liquid flow to show the operation of the fluidic OR logic gate. i–iii) Liquid flow sequence in the crack microvalves (box B, open) and iv−vi) the liquid flow in the wrinkle microvalves (box A, open) during a gradual releasing process. Reproduced with permission.^[^ ^283^ ^]^ Copyright 2021, American Chemical Society.

More recently, controlled wrinkling can be applied as an alternative patterning tool to fabricate microfluidics.^[^ ^282^, ^283^, ^284^, ^285^ ^]^ Liu et al. fabricated strain‐tunable crack and oriented wrinkle microvalves for microfluidic devices, as depicted in **Figure** **27**. The crack microvalves initially closed before stretching are opened as the tensile strain is applied, whereas the wrinkle microvalves exhibit the opposite trend. Both microchannels can be switched between the “on” and “off” states to serve as a microvalve by the applied tensile strains. These microfluidic devices have been demonstrated for microsphere screening (Figure 27b) and programmable microfluidic logic devices (Figure 27c).^[^ ^283^ ^]^

### 5.9 Prospective Applications

Other than those successful applications of oriented wrinkled interfaces depicted above, they have been employed for different purposes such as marine antifouling coatings in which fouling‐release and colonization prevention are achieved because of the surface topography.^[^ ^286^, ^287^, ^288^, ^289^, ^290^ ^]^ In addition, wrinkles have served as supports for facilitating transmembrane molecular transport while their connection with narrow interlayer channels can form a selective network.^[^ ^291^, ^292^ ^]^

The catalysis field has also provided an application platform for oriented wrinkled surfaces. They have served as a new smart catalytic reactor to trigger a reaction in built‐in microchannels with suppressing catalyst leaching while accelerating reaction kinetics by both nanoconfinement and photothermal effect.^[^ ^293^, ^294^ ^]^

## 6 Conclusions

Since the initial report of oriented wrinkle formation employing soft foundations in the late 1990s, there has been an increasing emphasis on fabricating oriented wrinkled surfaces in a controlled manner. As described in this review, these surfaces can currently be fabricated through diverse approaches based on the chemical structure of the precursor films (layered, homogenous, or gradient), as a function of the instability involved (temperature, mechanical pretension, osmotic pressure) in the wrinkle formation and as a function of applying anisotropy in the orientation control. Consequently, the approach employed defines not only the wrinkle characteristics but also the resulting surface chemical composition and topography that can be extensively varied.

By virtue of their fabrication universality, low fabrication costs, and capacity for scale‐up fabrication, these surfaces are widely employed in diverse fields. Among others, the distinctive properties of oriented wrinkled surfaces (such as flexibility, reversible variations in dimensions, or periodicity) provide the basis for their employment in fabricating templates for directing particle organization, flexible electronic devices, surfaces with controlled wettability, supports with tunable opticity or adhesion, enhanced cell alignment, and microfluidic devices.

Despite the comparatively extensive work achieved to date toward their fabrication, novel wrinkling systems are constantly being developed optimizing the currently existing approaches to facilitate the fabrication procedure (i.e., one‐step fabrication) or producing platforms with applications in burgeoning fields such as marine antifouling coatings as well as smart catalytic reactor.

## Conflict of Interest

The authors declare no conflict of interest.
